# Testers' compliance to the quality standards of HIV rapid testing services in Primary Health Care Facilities in Makete District, Tanzania

**DOI:** 10.4314/ahs.v22i2.6

**Published:** 2022-06

**Authors:** Ruth Raymond Ngowi, Gasto Frumence

**Affiliations:** 1 Health Quality Assurance Unit, Ministry of Health, Community Development, Gender, Elderly and Children, P. O. Box 743, Dodoma, Tanzania; 2 School of Public Health and Social Sciences, Department of Development Studies, Muhimbili University of Health and Allied Sciences, P. O. Box 65454, Dar es Salaam, Tanzania

**Keywords:** HIV rapid testing, quality standards, non-laboratory testers, compliance to quality standards, primary health care facilities

## Abstract

**Background:**

HIV rapid testing services is one among key interventions in the controlling of HIV/AIDS. Despite availability of quality standards, the quality of HIV rapid testing services remains questionable since non-laboratory testers are allowed to conduct testing while they are not specialized in providing testing services.

**Objective:**

To evaluate the compliance to the quality standards of HIV rapid testing services provided by non-laboratory testers in Makete District, Tanzania

**Methods:**

An explanatory descriptive study employing quantitative approach of data collection was used. An observation of 23 non-laboratory testers performing HIV rapid tests, observation of HIV testing points and documents review was done in 23 testing points to collect data. Data were analyzed using a programmed excel sheet and a three-point scale was used to determine the level of compliance to quality standards.

**Results:**

Analysis shows that out of 23 testing points visited, the level of compliance to quality standards was lower for 22 (95.6%) testing points and moderate in 1 (4.4%) testing point. None of the testing point was highly complied to quality standards for HIV rapid testing services.

**Conclusion:**

The quality of HIV rapid testing services provided by non-laboratory testers is below the established quality standards for HIV rapid testing services.

## Background

HIV rapid testing services is among key interventions in the controlling of HIV/AIDS since it provides chances to access HIV care, treatment, prevention, and support services.^1^,^2^ HIV rapid testing services allow people diagnosed with HIV to be linked appropriately and on timely manner to treatment and care services.^3^,^4^ The World Health Organization (WHO) established quality standards for the implementation of HIV rapid testing services in order to ensure the quality of testing services.^5^ Such standards include: personnel training and certification; physical facility (such as room, hand washing facilities, waste management equipment, light source, cabinets for storage of equipment's and registers); and safety practices (such as availability and use of PPEs, availability and use of job aids and SOPs for safety as well as hand washing during provision of services). According to the WHO, other standards include: pre testing phase (availability of testing guidelines and algorithm, supplies for sample collection and proper client identification); testing phase (such as accurate routinely use of Quality Control specimen, use of SOPs, timer and sample collection devices during testing). Other standards for the implementation of HIV rapid testing services include: documentation and record keeping (proper use of standardized registers to document test results, patient's information and use of test kits); participation in External Quality Assessment program that involves supportive supervision, participation in Proficiency Testing (PT), reviewing and implementing corrective action in case of unsatisfactory PT results.^5^

Several studies have been conducted to evaluate the quality of HIV rapid testing services. An auditing study conducted in Caribbean and African countries, which involved 968 HIV testing points demonstrated that 20% of the testing points scored level 0; 46% of the testing points scored level 1; 31% of the testing points scored level 2; while 3% of the testing points scored level 3 and none of the testing point that had high level of compliance to quality standards such that none of the testing points scored level 4.^6^

Furthermore a study conducted in 102 private health facilities in Kampala Uganda to evaluate the quality of HIV rapid testing services demonstrated that the quality of HIV rapid testing services is below the recommended quality standards.^1^ In addition, the study found that 34% health facilities did not have enough space for storage of HIV test kits; only 50% health facilities had and used HIV Counseling and Testing guidelines; 27% providers had never received in service training in HIV Counseling and Testing; 61% health facilities did not have SOPs for HIV rapid testing and 61% of the testers had never received supportive supervision from higher level.^1^ A technical report on increasing accountability for reliability of HIV rapid test in 22 testing points in Rungwe District, Tanzania, demonstrated that there is noncompliance to quality standards during the provision of HIV rapid testing services whereas non-compliance to HIV rapid safety standards was 18%, non-compliance to HIV rapid testing standards was 17%, non - compliance to post testing and documentation standards was 14% and non - compliance to External Quality Assessment standards was 37%.^7^

Tanzania has also adopted the WHO quality standards for the implementation of HIV rapid testing services.^8^ Despite the significant role played by HIV rapid testing services in improving health outcomes, the quality of this services remains questionable since testing is conducted by non-laboratory professionals who are not specialized in providing laboratory services hence a need was identified to monitor quality of performance of testers and sites.^8^,^9^ Poor quality of HIV testing results have negative effects to clients, health services and community at large since it leads into wrong initiation or delay into initiation of treatment and may also lead into misuse of resources. 5,10,11 WHO stresses the need to ensure quality of HIV rapid testing services by conducting regular quality auditing of testing sites in order to determine the compliance to the quality standards.^5^ In other countries a number of evaluation studies have been conducted to evaluate the compliance to HIV rapid testing services quality standards. However, in Tanzania, since the adoption of quality standards for HIV rapid testing services, there are limited studies conducted to evaluate compliance to these quality standards leading to inadequacyof information regarding testers' compliance to quality standards of HIV rapid testing services of which this study was an attempt to fill this research gap.

## Methods

### Study settings and design

The study was conducted at Makete District Council in Njombe Region, Southern Tanzania. HIV Records for 2016/2017 show that Njombe Region has the highest HIV prevalence (11.4%) in Tanzania.^12^ The study was an explanatory descriptive study design, which employed quantitative approach to collect data on the levels of compliance to the quality standards in the implementation of HIV rapid testing services. The study population included one (1) non-laboratory tester available at each point of care on the day of the visit, making a total of twenty-three (23) non-laboratory testers. Twenty-three (23) HIV testing points available at the six (6) selected Primary Health Care facilities (both Public and Private) in Makete District were included in the study.

### Sampling and procedures

Purposive sampling technique was used to select both public and private health facilities where by all HIV rapid testing points were studied and non-laboratory testers who provided services on the day of the study were observed when performing actual HIV rapid tests.

An on-site checklist for document review and observation adopted from WHO by the Tanzania Ministry of Health was used to collect quantitative data. The main areas of observations included: personnel training and certification; physical facility; safety; pretesting phase; testing phase; documentation and records as well as external quality assessment.

Ethical clearance to conduct this study was obtained from Research and Ethics Committee (REC) of Muhimbili University of Health and Allied Sciences (MUHAS). Permission to conduct the study was requested from the District Medical Officer of Makete District and officer in charges of the respective health facilities. Study participants were informed about the purpose of the study, procedures, risk and benefits for them to participate in the study. Informed written consent was obtained from each study participant after agreeing to participate in the study.

### Data management and analysis

Data were collected manually and entered into excel sheets and they were cleaned and validated. All descriptive data were analyzed using programmed excel sheets and data were presented in frequency tables of numbers, percentages (for each quality standard) and level of compliance to quality standards for HIV rapid testing service. Summation of each response of the open ended question in each quality standard was used to determine the average compliance for each quality standard. A three-point scae (high, moderate and lower) was used to determine the level of compliance to all quality standards for HIV rapid testing services for each testing points whereas testing points scored level 4 (score of 90% or higher) were defined as highly complied to quality standards; level 3 (score of 80% – 89%) were defined as moderately complied to quality standards; level 2 (60% – 79%), level 1 (40% – 59%) and level 0 (less than 40%) were defined as lower complied to quality standards.

## Results

### Social demographic characteristics of study population

Most of the HIV testers (34.8%) worked on wards, Reproductive and Child Health section (21.7%) and Care and Treatment Centre (13%). The majority of the testers (52%) were from the district level health facilities while about 22% of them were from dispensaries. Majority of the testers (78%) worked in the government health facilities and the remaining 22% worked in private health facilities. Most of testers were women (78.3%) while 21.7% were men. All testers (100%) had ordinary level of secondary education whereas among them 69.6% testers had certificate level of education and 30.4% had diploma level of education. Only one study participant (4.4%) was clinical officer, 30.4% were enrolled nurses and 34.8% were Medical attendants while 30.4% were assistant nursing officers.

### Level of compliance to HIV rapid testing quality standards

**Figure 1:**
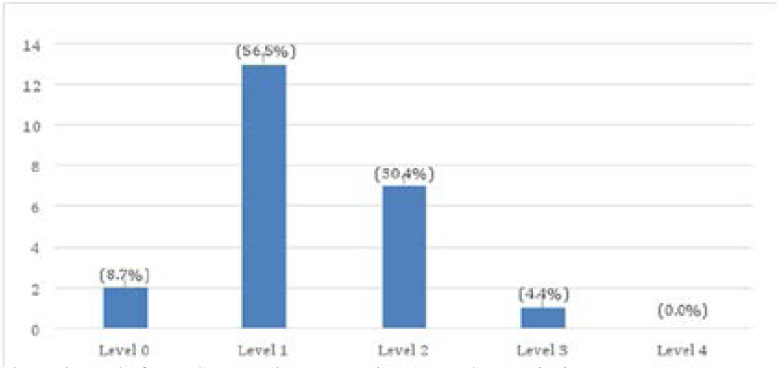
Level of compliance to the HIV rapid testing quality standards among 23 testing points

Evaluation results show that out of 23 testing points visited, the level of compliance to the quality standard was lower for 22 (95.6%) testing points. Two (2) testing points (8.7%) scored level zero, 13 (56.5%) testing points scored level one and 7 (30.4%) scored level two. The level of compliance to HIV rapid testing quality standards was moderate in 1 (4.4%) testing point. None of the testing point had high level of compliance to the HIV rapid testing quality standards, meaning that no testing point attained level four. Figure 1 shows the level of compliance to the HIV rapid testing quality standards for the 23 testing points.

**Figure 2:**
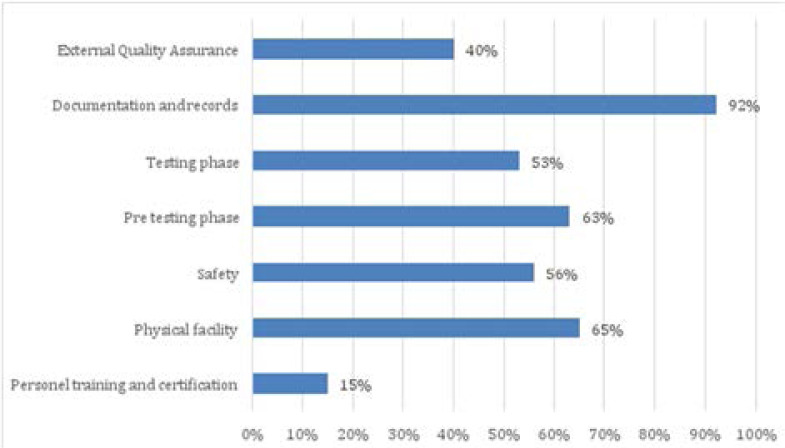
Average compliance for each HIV rapid testing quality standards

Average compliance for each quality standard was also determined by observing how non-laboratory testers and testing points operations comply with such standards. These standards include: personnel training and certification; physical facility, safety, pretesting phase, testing phase, documentation and records as well as external quality assessment. Figure 2 shows the average compliance for each HIV rapid testing quality standard.

### Personnel Training and Certification (Competence of testers)

The average compliance to personnel training and certification quality standards was 15% whereas: only 9 (39%) out of 23 non laboratory testers that provided HIV rapid testing services on the day of the study received comprehensive training on HIV rapid testing using the nationally approved curriculum; only 2 (9%) non-laboratory testers were trained on external quality assessment (EQA)/proficiency testing (PT) process; only 5 (22%) non-laboratory testers were trained on safety and waste management procedures and practices. No any non - laboratory tester has been certified or licensed through a national certification program to perform HIV rapid testing services.

### Physical Facility

Average compliance to physical facility quality standards was 65% whereas: 16 (66%) testing points have designated area for providing HIV rapid testing services; 11 (48%) testing points were clean and organized for testing; 22 (96%) of testing points had sufficient lighting; none of the testing points store test kits in temperature-controlled environment and there was no device to monitor temperature in all visited testing points and 18 (78%) testing points had sufficient and secure storage space for test kits and other testing consumables.

### Safety

Average compliance to safety standards was 56% whereas: only 2 (9%) testing points had SOPs to manage spills of blood and other body fluids; only 4 (17%) testing points had SOPs to address accidental exposure to potentially infectious body fluids through a needle stick injury, splash or other sharps injury; all 23 (100%) testing points had personal protective equipment (PPE) and testers properly used PPEs through the testing process; 13 (57%) testing points had hand-washing facilities such as water and liquid soap. Only 6 (26%) testing points handled properly waste materials such as sharps, infectious and non-infectious waste and only 13 (56%) testing points disinfect working area with the appropriate disinfectant such as chlorine.

### Pre testing Phase

The average compliance to pre testing phase standards was 63% whereas: only 9 (39%) testing points had national guidelines for HIV testing services; all 23 (100%) testing points use national algorithm and approved HIV rapid kits; expired HIV rapid test kits such as uni-gold were available in 9 (39.1%) testing points; only 4 (17%) testing points had a standard process for stock management; only 4 (17%) testing points had posted job aides on client sample collection; 19 (82%) testing points had sufficient supplies for client sample collection and in only 9 (39%) testing points, client's identifiers were recorded in the HIV testing register and on test devices as per national guidelines

### Testing Phase

The average compliance for testing phase standards was 53% whereas: only 5 (54%) testing points had SOPs on HIV testing procedures and posted at the testing area; timers were available and used routinely in only 1 (4.3%) testing point; sample collection devices such as capillary tube, loop, disposable and pipettes were used accurately in 22 (95%); testing procedures such as correct use of buffer and reading of results on time were adequately followed in 16 (70%) testing points and positive and negative quality control specimens were routinely used in 13 (57%) testing points. Retest for verification was done in 4 (67%) health facilities out of 6 health facilities visited.

### Documentation and records

Average compliance to documentation and records standards was 92% whereas: 20 (87%) testing points had standardized register for documentation, which contain key elements in the registers such as client demographics, kit names, lot numbers, expiration dates and tester name. The final HIV results were recorded correctly in 16 (70%) testing points; client documents and records were securely kept throughout all phases of the testing process in 22 (95%) testing points.

### External Quality Assessment

Average compliance to External Quality Assessment was 40% whereas: 11 (48%) testing points were enrolled in Proficiency Testing (PT) program; out of 11 testing points enrolled into PT program, only 4 (36%) testing points implement corrective action in case of unsatisfactory PT results; 21 (91%) testing points received periodic supervisory visit and written supervision report was available in only 1 (5%) testing point.

## Discussion

This study has shown that the level of compliance to the quality standards for HIV rapid testing is below the recommended standards. Majority of observed HIV testing points scored level one and none of the testing point was highly complied to quality standards, meaning that no testing points scored level four. The results of this study are similar with the study conducted in Kampala Uganda to assess the quality of HIV counseling and testing services, which demonstrated that the quality of HIV testing services is below the recommended standards.^1^ Similarly a study conducted in Africa and Caribbean countries where 1740 HIV testing points were audited reported similar findings showing that majority of HIV testing points scored level 1 and none of the testing point was highly complied to quality standards for HIV rapid testing services.^6^ On the contrary, a study done in South African to evaluate the quality of HIV rapid testing services in 11 clinics reported different findings from our study indicating that majority of the HIV testing points (90.9%) were moderately complied to the WHO quality standards for testing services. It was highlighted that the moderate performance of these testing points in South Africa was due to much work that have been done to improve the quality management system in these testing points.^13^

In this study, the highest average compliance score was seen on documentation and records followed by physical facility that included availability of conducive designated areas for provision of HIV rapid testing services and the lowest average compliance score was seen on personnel training and certification, followed by External Quality Assessment, testing phase, safety and pre testing phase. In addition, this study revealed that availability of new National HIV register that require all key elements to be documented in one register has contributed to improved documentation and record keeping for different data hence results into higher score for documentation and records quality standards. Furthermore, this study shows that most of the non-laboratory testers did not receive comprehensive training to perform HIV rapid testing services and none of them were certified/licensed to perform HIV rapid test through the National Certification Program. This situation has resulted into the lower score for the personnel training and certification quality standards, which was below the recommended quality standards for HIV rapid testing services, which requires that services should be provided by testers who received comprehensive training as well as evaluated for their competence and certified to provide testing services.^2^,^14^,^15^ Results of this study are comparable to the study conducted in Africa and Caribbean countries in 2015 whereas 968 HIV testing points were audited against the pre-established quality standards for HIV rapid testing services, the study reported that highest average compliance score were also obtained on documentation and records, and physical facility. 6 Furthermore, these results are comparable with the results on assessment of compliance to HIV rapid testing quality standards conducted in Tanzania in 2016 whereby the compliance to quality standards was lower for personnel training and certification, External Quality Assessment, testing phase and safety standards.^7^

In this study we observed low compliance to the quality standards for External Quality Assessment (EQA) and about half of the testing points were enrolled into proficiency testing program and most of the testers were not trained to perform samples for proficiency testing. A study conducted in South Africa to evaluate the quality management system for HIV rapid testing services reported similar findings indicating that the compliance to EQA standards was low and only two clinics out of eleven clinics were enrolled into proficiency testing program. 13 Another study conducted in Africa and Caribbean countries show dissimilar results as compared to this study whereas 968 HIV testing points were audited against the pre-established WHO quality standards for HIV rapid testing services. The study reported that there was moderate (74%) compliance to EQA standard, which was due to periodic supportive supervision conducted, increased number of enrollment of testing points into proficiency testing as well as training of staff on how to perform samples for proficiency testing.^6^

In this study, non-adherence to testing procedures including the use of small volume of buffer and non-adherence on time to read result whereby timers were not used during testing were observed. Similar findings were reported in a study conducted in Zambia National Reference Hospital in which the use of inadequate volume of specimen and use of incorrect volume of buffer leading into poor quality of test results was reported.^16^ Our study findings are also similar to a systematic review of 64 studies from Africa, America, Asia, Europe other multi-country which was done to evaluate the quality of HIV rapid testing services and misdiagnosis of HIV status. They reported that there were suboptimal testing strategies during testing, which led into poor adherence of testing procedures that include reading of results too early before the optimal time as a result of lack of timers and using of inappropriate amount of reagents.^11^

Our study has shown that a few testing points had guidelines for HIV testing services and Standard Operating Procedures (SOPs). These guidelines and SOPs are important documents for providing guidance and procedures needed to be followed during testing hence lack of guidelines and SOPs compromises the quality of HIV rapid testing services.^17^,^18^ These results are comparable with the study conducted to assess the quality of HIV counseling and testing services in Uganda which showed that only 25% of the audited facilities have guidelines for HIV testing and only 39% of the facilities have SOPs for HIV rapid testing services hence testers do not have any reference to make in case they need to do so during testing.^1^ Similar findings from another study conducted in South Africa to evaluate the quality of HIV rapid testing services, reported that 39.1% of the testers do not have SOPs for HIV testing and only 5 (16%) testing points had guidelines for HIV rapid testing services.^19^

## Limitation of the study

Some of the testing points were temporarily closed during data collection period due to lack of trained testers to provide service, and probably such testing points could have useful information regarding the practice of implementation of HIV rapid testing services in studied facilities. Furthermore, due to resource constrains only six facilities were studied, which might have limited the data collected. However, we believe that this study provides valuable insights regarding the quality of HIV rapid testing services provided by non-laboratory testers in the rural setting district in Tanzania.

## Conclusion

The quality of HIV rapid testing services provided by non-laboratory testers in Makete District is below the established quality standards set for provision of HIV rapid testing services. Most of the non-laboratory testers were not trained, evaluated for their competencies and were not certified or licensed to provide HIV rapid testing services. Some of HIV rapid testing points do not have Guidelines and SOPs that guide provision of HIV rapid testing services, which compromises the quality of HIV testing services. The Ministry of Health and Management of the health facilities should invest more efforts in training and strengthening of certification of non-laboratory testers, increasing enrolment of testing points into proficiency testing and strengthening supervision of testing points in order to improve compliance to quality standards.
